# Genetic Characterization of the Influenza A Pandemic (H1N1) 2009 Virus Isolates from India

**DOI:** 10.1371/journal.pone.0009693

**Published:** 2010-03-15

**Authors:** Varsha A. Potdar, Mandeep S. Chadha, Santosh M. Jadhav, Jayati Mullick, Sarah S. Cherian, Akhilesh C. Mishra

**Affiliations:** National Institute of Virology, Pune, India; Institute of Infectious Disease and Molecular Medicine, South Africa

## Abstract

**Background:**

The Influenza A pandemic H1N1 2009 (H1N1pdm) virus appeared in India in May 2009 and thereafter outbreaks with considerable morbidity and mortality have been reported from many parts of the country. Continuous monitoring of the genetic makeup of the virus is essential to understand its evolution within the country in relation to global diversification and to track the mutations that may affect the behavior of the virus.

**Methods:**

H1N1pdm viruses were isolated from both recovered and fatal cases representing major cities and sequenced. Phylogenetic analyses of six concatenated whole genomes and the hemagglutinin (HA) gene of seven more isolates from May-September 2009 was performed with reference to 685 whole genomes of global isolates available as of November 24, 2009. Molecular characterization of all the 8 segments was carried out for known pathogenic markers.

**Results:**

The first isolate of May 2009 belonged to clade 5. Although clade 7 was the dominant H1N1pdm lineage in India, both clades 6 and 7 were found to be co-circulating. The neuraminidase of all the Indian isolates possessed H275, the marker for sensitivity to the neuraminidase inhibitor Oseltamivir. Some of the mutations in HA are at or in the vicinity of antigenic sites and may therefore be of possible antigenic significance. Among these a D222G mutation in the HA receptor binding domain was found in two of the eight Indian isolates obtained from fatal cases.

**Conclusions:**

The majority of the 13 Indian isolates grouped in the globally most widely circulating H1N1pdm clade 7. Further, correlations of the mutations specific to clade 7 Indian isolates to viral fitness and adaptability in the country remains to be understood. The D222G mutation in HA from isolates of fatal cases needs to be studied for pathogenicity.

## Introduction

The first influenza pandemic of the 21st century was declared with the emergence of a novel Influenza A (H1N1) strain in Mexico and the USA in April 2009 [1]. The virus has been detected in about 207 countries infecting more than 622,482 people worldwide with more than 7,820 deaths as of November 22, 2009 [2]. The virus was first detected in India in May 2009 [3]. Since then outbreaks have been reported from many parts of the country. As of December 6, 2009, the total number of confirmed cases in India was 19,632 with 621 deaths [4].

Several reports describe both the emergence and the pandemic potential of the virus in the perspective of prior pandemic influenza viruses of 1918 (H1N1), 1957 (H2N2) and 1968 (H3N2) [5], [6] by comparison of the available genetic sequence data. The genetic analysis of the novel H1N1 virus isolated from a patient in California revealed that it was a recent reassortant of gene segments from both the North American and Eurasian swine lineages [7]. It was determined that the virus possesses the polymerase basic-2 (PB2) and polymerase A (PA) genes of North American avian virus origin, the polymerase basic-1 (PB1) gene of human H3N2 virus origin, the hemagglutinin (HA), nuclear protein (NP) and non-structural (NS) genes of classical swine origin and the neuraminidase (NA) and matrix (M) genes of Eurasian swine virus origin. The human-like PB1 gene and avian-like PB2 and PA genes however have been circulating in pigs since 1997–98 in the form of a triple reassortant swine virus [8]. Certain specific molecular markers predictive of adaptation to human were found to be absent in the pandemic H1N1 2009 (H1N1pdm) viruses suggesting that, previously unrecognized molecular determinants could be responsible for the transmission among human [7]. Other reports comparing the HA gene sequence with those of the earlier influenza pandemics have shown that human-specific markers supporting efficient transmissibility of these viruses in human are present in the H1N1pdm virus [9], [10]. The amino acids in the active site of NA suggest susceptibility [7] to Oseltamivir and Zanamivir type inhibitors, though, in view of the extensive use of these antivirals the emergence of drug-resistant variants is a matter of serious concern. Further, continuous monitoring of the evolution of this virus is advocated to track the mutations that may increase pathogenicity and/or transmissibility.

A recent study [11] revealed that the early diversification of the H1N1pdm virus based on concatenated whole genomes resulted into seven lineages, clade 1–7, that showed defined spatial patterns. Understanding the virus evolution within India in relation to global diversification of the virus is also essential. In this study, we present the analysis of whole genome sequences of six Indian isolates and the HA gene sequences from another seven isolates for genetic characterization and comparison with global isolates.

## Results

The first isolate from India (A/India-Hyd/NIV51/2009) was from a traveler reaching Hyderabad on May 13, 2009 from the USA. Positive cases of H1N1pdm virus were thereafter detected from major cities (Pune, Delhi, Mumbai, Chennai and Bangalore) with maximum fatality reported from Pune and Bangalore [4]. Selected samples were processed for virus isolation. Isolates representing different geographical regions, disease severity and time points between May-September 2009 were sequenced (Table 1).

**Table 1:** Details of patients and H1N1pdm viruses isolated during May–September 2009.

Sr No.	Name of the Isolate	Date of Collection	Location of the patient	Age (yr/mth)	Sex	Status of patient	Genes sequenced
1	A/India-Hyd/NIV51/2009	13 May 2009	Hyderabad	23 yr	M	Recovered	PB2, PB1, PA, HA, NP, NA, M, NS
2	A/India-Blore/NIV236/2009	26 June 2009	Bangalore	2.5 yr	M	Recovered	PB2, PB1, PA, HA, NP, NA, M, NS
3	A/India-Blore/NIV310/2009	01 July 2009	Bangalore	9 yr	F	Recovered	PB2, PB1, PA, HA, NP, NA, M, NS
4	A/India-Pune/NIV6196/2009	16 August 2009	Pune	17 yr	M	Death	PB2, PB1, PA, HA, NP, NA, M, NS
5	A/India-Pune/NIV6447/2009	17 August 2009	Pune	22 yr	F	Death	PB2, PB1, PA, HA, NP, NA, M, NS
6	A/India-Pune/NIV8489/2009	22 August 2009	Pune	42 yr	F	Death	PB2, PB1, PA, HA, NP, NA, M, NS
7	A/India-Delhi/NIV3610/2009	13 August 2009	Delhi	12 yr	M	Recovered	HA
8	A/India-Mum/NIV5442/2009	16 August 2009	Mumbai	2 mth	F	Death	HA
9	A/India-Pune/NIV9355/2009	29 August 2009	Pune	20 yr	M	Death	HA
10	A/India-Mum/NIV9945/2009	3 Sept. 2009	Mumbai	NA	M	Death	HA
11	A/India-Pune/NIV10278/2009	7 Sept. 2009	Pune	6 yr	M	Death	HA
12	A/India-Delhi/NIV3704/2009	8 Sept. 2009	Delhi	13 yr	M	Recovered	HA
13	A/India-Pune/NIV10604/2009	10 Sept. 2009	Pune	3 yr	F	Death	HA

### Phylogenetic analysis

Sequence analysis of the whole genome of the six Indian isolates revealed >99% nucleotide identity with the A/California/04/2009 (H1N1) prototype strain in all the gene segments. Similarly, 99.06% amino acid identity was noted in the HA of the Indian isolates with respect to A/California/04/2009. The percent amino acid divergence (PAD) within each gene segment of the six Indian isolates ranged from 0% (in PB2 and M) to 0.66% (in HA) while the PAD within the HA genes of the 13 isolates was 0.58±0.17%.

**Figure 1:**
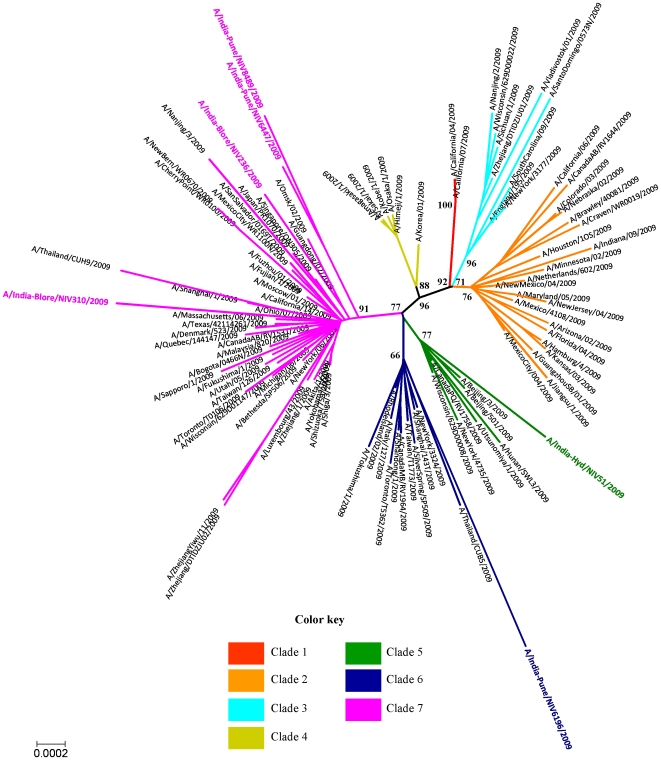
Phylogenetic tree of concatenated whole genomes of 6 Indian isolates and representative global isolates using the Neighbor-joining method. Scale bar indicates number of nucleotide substitutions per site.

Phylogenetic analysis of the six concatenated whole genome sequences was performed along with the whole genomes of global isolates available in the GenBank. In an earlier concatenated whole genome analysis of 240 H1N1pdm virus isolates up to July 2009 [11], seven discrete clades of the H1N1pdm viruses circulating globally were observed. In the present study, the analysis was performed with an enhanced data set (n = 685) as of November 24, 2009. Clade 1 included amongst others the California/04/2009 and California/07/2009 isolates while Clade 2 included isolates from Mexico, California, Canada, France, Germany, Netherlands, China and multiple US states; clade 3 - France, England, Russia, China, Canada, Mexico and the US states; clade 4–two east Asian countries, Korea and Japan; clade 5–Canada, China, Japan, the USA (mainly Wisconsin isolates) with an addition of Thailand and clade 6–Canada, Italy, China, Japan with new additions of Germany, Taiwan, Thailand and few US states. The clade 7 which was the largest clade included isolates from Canada, China, Japan, Germany, Italy, Luxembourg, Russia and several states of the USA with Taiwan, Denmark, Singapore, Malaysia, Central and South America getting added. Figure 1 depicts the concatenated whole genome phylogeny based on 96 isolates representative of different geographical regions along with the genomes of the Indian isolates.

As seen in figure 1 the Indian isolates belonged to three different clades. The earliest Indian isolate (A/India-Hyd/NIV51/2009) of May 2009 clustered with clade 5 isolates, one Indian isolate (A/India-Pune/NIV6196/2009) of mid August belonged to clade 6 while four Indian isolates (A/India-Blore/NIV236/2009, A/India-Blore/NIV310/2009, A/India-Pune/NIV6447/2009, A/India-Pune/8489/2009) of the period from June end to August 2009 clustered into clade 7.

**Figure 2:**
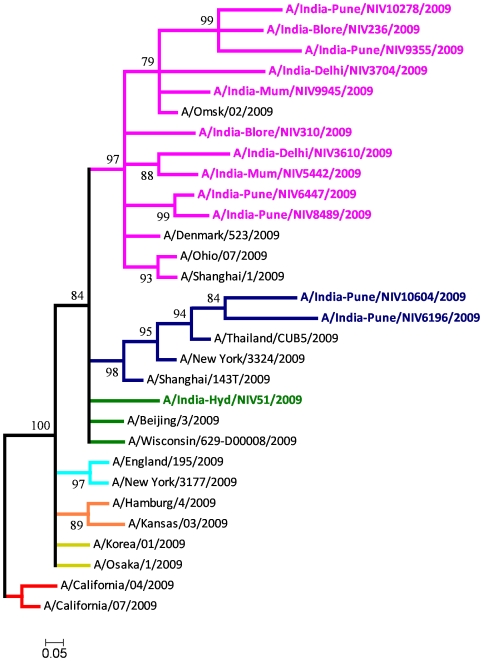
HA gene phylogeny of 13 Indian isolates and representative global isolates using the Bayesian method. Scale bar indicates number of nucleotide substitutions per site. The color scheme follows that of Figure 1. Scale bar indicates number of nucleotide substitutions per site.

HA-based phylogenetic analysis was also performed for the 13 Indian isolates with reference to the HA genes of the 685 global whole genomes. Though the clade assignment is based on concatenated whole genomes, notably in the HA gene phylogeny, the topology of the clades 6 and 7 was maintained. However, some minor changes were observed in the topology of the other clades. Figure 2 shows the HA-based Bayesian phylogeny considering the 13 Indian isolates and a few representative isolates of other clades. Except for A/India-Hyd/NIV51/2009, all the other Indian isolates clustered into clades 6 and 7 with strong bootstrap support. The A/India-Pune/NIV6196/2009 isolate maintained its position in clade 6 and so also the other four isolates retained their positions in clade 7. Of the seven additional Indian isolates, one isolate of September (A/India-Pune/NIV10604/2009) belonged to clade 6 while the remaining six isolates (A/India-Pune/NIV9355/2009, A/Delhi/NIV3610/2009, A/India-Mum/NIV5442/2009, A/India-Mum/NIV9945/2009, A/India-Pune/NIV10278/2009, A/India-Delhi/NIV3704/2009) of the period from August to September 2009 clustered into clade 7. No spatial or temporal patterns within the Indian isolates of clade 7 could be observed.

Mutations noted in the Indian isolates with respect to the prototype isolate (A/California/04/2009) are shown in Tables 2 and 3. The clade specific mutations [11] in different genes, NP: V100I; NA: V106I and N248D; HA: K-15E (K2E), Q293H (Q310H) and S203T (S220T); NS1: I123V, were also noted amongst the Indian isolates. The residue position within parenthesis for the HA is the numbering considered inclusive of the signal peptide. Additionally, mutations P83S (P100S) and I321V (I338V) in HA along with P224S in PA present in the Indian isolates have been observed in all the non-clade1 isolates [11]. Mutations S451N and I547T in the HA, T232P in the PB1 were unique to Indian isolates, the significance of which need to be addressed. The PB2 region of all the Indian isolates did not have any mutation.

**Table 2:** Mutations in the gene segments of the six whole genomes of H1N1pdm Indian isolates with respect to A/California/04/2009.

Gene segment	Residue Number	A/California/04/2009	Clade 5	Clade 6	Clade 7			
			A/India-Hyd/NIV51/2009	A/India-Pune/NIV6196/2009	A/India-Blore/NIV236/2009	A/India-Blore/NIV310/2009	A/India-Pune/NIV6447/2009	A/India-Pune/NIV8489/2009
PB1	61	T	.	.	.	.	.	I
	254	F	L	L	.	.	.	.
PA	28	P	Q	.	.	.	.	.
	224	P	S	S	S	S	S	S
	271	P	.	R	.	.	.	.
	581	M	.	.	.	.	L	.
NP	100	V	I	I	I	I	I	I
	232	T	P	.	P	.	P	.
NA	30	I	V	V	V	.	V	.
	106	V	I	I	I	I	I	I
	189	N	.	S	.	.	.	.
	248	N	D	D	D	D	D	D
	256	F	.	.	V	.	.	.
NS1	28	G	.	S	.	.	.	.
	123	I	.	.	V	V	V	.
	154	G	.	.	.	.	.	R
NS2	115	A	T	.	.	.	.	.

**Table 3:** Mutations in the HA gene of 13 H1N1pdm Indian isolates with respect to A/California/04/2009.

Residue position In HA (without signal peptide)	A/California/04/2009	Clade 5	Clade 6		Clade 7									
		A/India-Hyd/NIV51/2009	A/India-Pune/NIV6196/2009	A/India-Pune/NIV10604/2009	A/India-Blore/NIV236/2009	A/India-Blore/NIV310/2009	A/India-Pune/NIV6447/2009	A/India-Pune/NIV8489/2009	A/India-Delhi/NIV3610/2009	A/India-Mum/NIV5442/2009	A/India-Pune/NIV9355/2009	A/India-Mum/NIV9945/2009	A/India-Pune/NIV10278/2009	A/India-Delhi/NIV3704/2009
−15	K	.	E	E	.	.	.	.	.	.	.	.	.	.
−13	I	.	.	.	.	.	T	T	.	.	T	.	.	.
−2	A	T	.	.	.	.	.	.	.	.	.	.	.	.
83	P	S	S	S	S	S	S	S	S	S	S	S	S	S
154	K	.	.	.	.	.	.	.	E	.	.	.	.	.
197	T	A	A	A	A	A	A	A	A	A	A	A	A	A
203	S	.	.	.	T	T	T	T	T	T	T	T	T	T
222	D	.	.	.	.	.	.	.	.	.	G	.	G	.
223	Q	.	.	.	R	.	.	.	.	.	.	.	.	.
234	V	.	.	.	.	.	.	I	.	.	.	.	.	.
293	Q	.	H	H	.	.	.	.	.	.	.	.	.	.
321	I	V	V	V	V	V	V	V	V	V	V	V	V	V
339	G	.	R	.	.	.	.	.	.	.	.	.	.	.
370	N	.	.	.	.	.	.	.	.	.	K	.	.	.
451	S	.	.	.	N	.	.	.	.	.	N	.	N	.
547	I	.	.	.	T	.	.	.	.	.	T	.	T	.

### Molecular characterization

HA of the Indian isolates, as in other H1N1pdm viruses possessed D190 in the receptor binding site, which is known to confer binding of H1 viruses to human receptors [12] probably, supporting the efficient transmissibility of these viruses in human [9]. The majority of Indian isolates, (11 of 13) as well as H1N1pdm viruses, possessed D225 [12] in the receptor binding domain (RBD), though two of the Indian isolates (A/Pune/NIV9355/2009, A/India-Pune/NIV10278/2009) had D225G (D222G as per H1N1pdm numbering). Notably, this mutation was observed in the isolates from two fatal cases of the later period (late August - and September). Among other critical residues [13] known to confer human specificity in H5 viruses, E227, P221 and E216 were also noted in H1N1pdm viruses including the Indian isolates. Key residues in the RBD [13] predicted to have a role in binding to human receptors (T98Y, S136T, 153W, 183H) were found to be Y98, S136, W153 and H183 in the Indian isolates as in other H1N1pdm viruses. The majority of H1N1pdm viruses, including the Indian isolates, had eight putative glycosylation sites at positions 27, 28, 40, 104, 293, 304, 498 and 557. They also possessed a single basic amino acid at the HA cleavage site instead of the polybasic cleavage site of highly pathogenic H5 and H7 viruses [14].

Mapping of the HA mutations observed in the Indian isolates (Table 3) on to the known antigenic sites of H1 [15] and H3 [16] was carried out. Mutation P83S observed in all the Indian isolates was located in the antigenic site E, while mutation K154E of A/India-Delhi/NIV3610/2009 was located in site B. Both the mutations D222G (A/India-Pune/NIV9355/2009, A/India-Pune/NIV10278/2009) and Q223R (A/India-Blore/NIV236/2009) were located in site D (vicinity of Ca1). Further, mutations T197A, Q293H and both S203T, V234I were found to be in the vicinity of site B (Cb), site C and site D (Ca1), respectively.

All the Indian isolates possessed residue H274 (position 275 in N1 numbering) a known marker for sensitivity to the neuraminidase inhibitor, Oseltamivir [17]. The H1N1pdm viruses including the Indian isolates had the genetic marker S31N in the M2 gene [18] suggesting Amantadine resistance. In the PB2 gene, the Indian and all other isolates of H1N1pdm possessed the low pathogenic markers, E627 [19] and D701 [20]. The PB1-F2 protein, which has been associated with the increased pathogenicity of the 1918 H1N1 virus and other highly pathogenic H5N1 viruses [21], is truncated in all H1N1pdm viruses, and is so also in the Indian isolates, due to stop codons at positions 12, 58 and 88. In the NS1 protein, the mutation D92E known for high virulence in human [22] is absent in all H1N1pdm viruses, as well as the Indian isolates. The PDZ ligand domain at the C-terminus of NS1 implicated in pathogenicity of the 1918 H1N1 virus [23] is absent, as all the H1N1pdm viruses and the Indian isolates had a truncated NS1 due to the presence of a stop codon at position 220.

A recent study [10] revealed that out of the 47 signatures that separate the avian viruses from human viruses by their non-glycoproteins, eight (PB2- A271; PB1- I336; PA- R356, N409; NP- I33, V100, K305, K357) were human-like in the H1N1pdm viruses. The Indian isolates showed all these signatures except V100 in NP.

## Discussion

Diversity of the Indian isolates at the amino acid level with respect to the prototype strain and within the Indian isolates was found to be maximum in the HA gene. In the concatenated whole genome-based phylogenetic analysis, with the enhanced data set in this study, no diversification beyond the existing seven clades [11] was observed. The two mutations in HA, K-15E and Q293H specific to clade 6 isolates and one mutation S203T specific to clade 7 isolates was adequate to delineate these two clades in the HA-based phylogeny. Phylogenetic analysis of the six whole genomes from India demonstrated that the earliest isolate of May 2009 from Hyderabad was a clade 5 isolate. This correlates to the time during which the global dissemination of the pandemic H1N1 virus was noted in Asian countries [11]. A movement of clade 5 virus was reported in Asia during week 8 (week 1 beginning April 1, 2009, the date of collection of the first H1N1pdm isolate). Notably, our first isolate represents an introduction of the virus in week 7. Incidentally, there were no isolates belonging to clade 5 during the weeks 9–11 in any Asian country. The observation that no other Indian isolate considered in this study belonged to this clade suggests that either the propagation of this strain was curtailed due to control measures or the fitness of the clade itself was questionable. Two isolates of India of the period August-September from Pune belonged to clade 6, both of which were from fatal cases. Though both these cases were not directly associated with any foreign travel history, it cannot be determined whether the clade evolved within the country or was again introduced into the country. All the other 10 Indian isolates belonged to clade 7 which has been the predominantly circulating clade since week 9 in Asia [11]. Two of our earliest isolates belonging to this clade of week 13–14 (June end–July 1) namely A/India-Blore/NIV236/2009 and A/India-Blore/NIV310/2009 from Bangalore had a history of foreign travel and thus may be indicative that the clade 7 was introduced from an external source. The isolates of six fatal cases in this study belonged to clade 7, though none were associated with the known markers indicative of increased pathogenicity in any of the genes.

The D222G mutation observed in two of the Indian isolates of clade 7 was also noted in twelve other H1N1pdm global isolates in GenBank at different time points during April–September, 2009. As reported earlier [10], [13], the two variants D and G at this position were also observed during the 1918 pandemic. Glycan microarray studies [13] have shown that HA possessing D190 along with G225 (G222 in H1N1pdm numbering) had specificity for both alpha 2, 3 and alpha 2, 6 linkages with no difference in virulence or transmissibility. Although the D222G mutation in the Indian isolates was from fatal cases, there were six other fatal cases having D222. Thus, the significance of this mutation in terms of pathogenicity needs to be verified.

Among the established pathogenic markers, no significant change was observed in the Indian isolates when compared to other H1N1pdm viruses. Of the mutations in HA, several of the changes were at or in the vicinity of the known antigenic sites and may therefore be of antigenic significance and have subsequent implications to vaccine efficacy. Further, the importance of the specific mutations observed in clade 7 and also in the Indian isolates needs further attention.

Overall, the analysis carried out in this study indicated that the dominant H1N1pdm lineage in India is clade 7, though both clades 6 and 7 are in co-circulation. Whether the predominance of clade 7 in India is related to the fitness and adaptability of the virus and more efficient human transmissibility requires further investigation in order to be understood.

## Materials and Methods

The clinical materials (throat and nasal swabs) obtained from patients were inoculated in specific pathogen-free (SPF) embryonated White Leghorn chicken eggs and Madin Darby Canine Kidney (MDCK) cell lines for isolation of the virus [24], [25]. Tissue culture fluid or allantoic fluid was harvested after observing inoculated eggs/MDCK cell line for cytopathic effect. All the samples were processed in the enhanced Biosafety level (BSL-2+) laboratory. Hemagglutination and Hemagglutination Inhibition (HAI) tests were performed using Guinea pig and fowl RBC as described earlier [26].

RNA was extracted from 140 µl of tissue culture fluid or allantoic fluid of each sample using QIAmp viral RNA mini kit (Qiagen, Germany) according to the manufacturer's specifications. One step RT-PCR was carried out to amplify all the eight segments using the recommended WHO-CDC whole genome primers [27]. Each of the segments were amplified in three to four fragments of 400 to 550 bp with 100 bp overlap in order to get at least four fold sequence coverage. To amplify each segment, 5 µl of RNA was added to 50 µl of master mix containing 25 µl of 2X enzyme buffer, 2 µl Invitrogen Superscript III enzyme, 1 µl of each reverse and forward primers, 1 µl RNasin (Promega, USA) and 15 µl molecular grade water. The reaction conditions were reverse transcription at 55°C for 40 min, initial denaturation at 94°C for 3 min followed by 35 cycles of 94°C for 30 sec, 50°C for 1 min, 68°C for 1 min with a final extension at 68°C for 5 min.

The resulting amplicons for all the eight segments were analyzed by 1% agarose gel electrophoresis. The expected size products that appeared as single bands were purified directly using PCR purification kits (Qiagen), whereas using Gel extraction kit (Qiagen) in case of multiple bands. DNA sequencing was carried out using Big Dye terminator V 3.1 cycle sequencing ready reaction kit (ABI, Foster City, CA) together with corresponding internal primers (WHO full genome sequencing primers) which were designed denovo to ensure specificity for each sequence. Subsequently, any unincorporated labeled dNTP's were removed using Dye-X removal column purification kit (Qiagen). The sequencing was done on ABI 3730 DNA analyzer and sequences were curated using sequencing analysis version 5.3 and MEGA version 4 was used for sequence alignment.

Whole genome of six isolates representing 3 recovery cases (A/India-Hyd/NIV51/2009, A/India-Blore/NIV236/2009, A/India-Blore/NIV310/2009) with travel history from an endemic country and 3 fatal cases (A/India-Pune/NIV6196/2009, A/India-Pune/NIV6447/2009, A/India-Pune/NIV8489/2009) were sequenced. Additionally, the HA gene of seven other cases were sequenced which represent 2 recovered cases (A/India-Delhi/NIV3610/2009, A/India-Delhi/NIV3704/2009) and 5 fatal cases (A/India-Mum/NIV5442/2009, A/India-Pune/NIV9355/2009, A/India-Mum/NIV9945/2009, A/India-Pune/NIV10278/2009, A/India-Pune/NIV10604/2009). The GenBank accession numbers of the six whole genome sequences and 7 additional HA gene sequences of the period May–September 2009 are from GU292344-98.

For phylogenetic analysis, the eight segments of the six whole genomes of the Indian isolates were concatenated and compared with 685 concatenated whole genome sequences available in GenBank as of November 24, 2009. MEGA version 4 [28] was used for constructing neighbor-joining (NJ) trees using the Kimura's two-parameter distance model with 1000 bootstrap replicates. The percent nucleotide identity (PNI) and percent amino acid identity (PAI) values were calculated as pairwise p-distances. The HA-based phylogenetic tree was constructed using the NJ method and the tree topology was further confirmed by using the Bayesian approach as implemented in Mr Bayes 3.2 [29]. The General Time Reversible (GTR) + Invariable sites (I) model with gamma-distributed rate variation across sites and a proportion of invariable sites was specifically used with other parameters kept at default.

For molecular characterization, the mutations in all the eight gene segments of Indian isolates were compared to the established human pathogenic markers of H1/H5 viruses. The four antigenic sites in HA designated Sa, Sb, Ca1/Ca2 and Cb identified on the A/PR/8/34(H1N1) virus [15] as well as antigenic sites A, B, C, D and E in H3 [30], [31] mapped on H1 [16] were used for identifying the antigenic sites of H1N1pdm Indian isolates. The NetNGlyc 1.0 Server [32] was used for the prediction of putative glycosylation sites in HA.
